# Letter to the Editor in regard to the “Effects of COVID-19 on in-hospital cardiac arrest: incidence, causes, and outcome” by Roedl et al.”

**DOI:** 10.1186/s13049-021-00965-4

**Published:** 2021-11-03

**Authors:** A. D. Krieg, J. Duval-Arnould, H. Newton, B. D. Winters, E. A. Hunt

**Affiliations:** 1 1600 North Wolfe Street, Baltimore, MD 21287 USA; 2 550 N Broadway Ste 401, Baltimore, MD 21205 USA; 3 1800 Orleans St. Sheikh Zayed Tower, Baltimore, MD 21287 USA

Dear Editor,

We have read the retrospective article entitled "Effects of COVID-19 on in-hospital cardiac arrest: incidence, causes, and outcome" by Roedl et al. published in the *Scandinavian Journal of Trauma, Resuscitation and Emergency Medicine* (2021; 29(30)). We want to commend the authors for this informative study and make some contributions.

In the study results, sustained return of spontaneous circulation (ROSC) was defined as stable circulation for at least 20 min. Moreover, the rates of sustained ROSC were comparable across the two study periods (2019 Non-COVID-19 period: 77% vs. 2020 COVID-19 period: 83%) [1]. These rates hint at similar outcomes across the study periods; however, the survival rates to discharge from hospital during the Non-COVID-19 period and COVID-19 period, particularly the rates of the survival of the COVID-19 positive subcohort, would provide valuable clinical data. A study collecting retrospective data from a public New York hospital during similar time periods (Non-COVID-19 period: March 1st, 2019 to May 15th, 2019; COVID-19 period: March 1st, 2020 to May 15th, 2020) reported a lower ROSC during the COVID-19 period compared to the Non-COVID-19 period (36% vs. 56%) [2]. This outcome correlated to a significantly lower survival to hospital discharge during the COVID-19 period (3% vs. 13%). Additionally, survival among COVID-19 patients experiencing IHCA was even lower (2%).

As we finalize and prepare to publish our analysis of the impact of the COVID-19 pandemic on cardiac arrest survival rates within our institution, we believe that data on the survival to discharge from Roedl’s cohort would be invaluable for both comparative reasons as well as to better characterize the effect of sars-cov2 on overall resuscitation success. To this end, we respectfully request that both survival to discharge and 30-day survival from the Roedl study be published as an addendum or otherwise be made available.

## Data Availability

Data cited in the above letter to the editor can be found in the original paper by Roedl et al. (10.1186/s13049-021-00846-w) or in the second source by Miles et al. (10.1161/CIRCOUTCOMES.120.007303). The datasets supporting the conclusions of this article are included within the article.

## Abbreviations

COVID-19: Coronavirus disease 2019
ICU: Intensive care unit
ROSC: Return of spontaneous circulation
