# Supplementary material for: Letter to the Editor in regard to the “Effects of COVID-19 on in-hospital cardiac arrest: incidence, causes, and outcome” by Roedl et al.”
Source: Scand J Trauma Resusc Emerg Med. 2021 Nov 3;29:159. doi: 10.1186/s13049-021-00965-4 (PMC8564593; doi:10.1186/s13049-021-00965-4)
Supplement: Related texts

# Reply: Response—Letter to the Editor in regard to the “Effects of COVID-19 on in-hospital cardiac arrest: incidence, causes, and outcome" by Roedl et al.”

**Authors:** Kevin Roedl

**Affiliations:** grid.13648.38 0000 0001 2180 3484 Department of Intensive Care Medicine, University Medical Centre Hamburg-Eppendorf, Martinistraße 52, 20246 Hamburg, Germany

To the Editor,

We thank Krieg et al. for the interest in our study and for their valuable comments. We agree with Krieg et al. that both study periods provided an almost similar rate of return of spontaneous circulation (ROSC) (2019: 77% vs. 2020: 83%, *p* = 0.237) [3]. In our cohort 40/84 (48%) and 34/93 (37%) were discharged alive from hospital in the 2019 and 2020 period (*p* = 0.551), respectively. Furthermore 26/40 (65%) and 27/34 (79%) had a favourable neurological outcome at ICU discharge in the 2019 and 2020 period, respectively. According the small sub-cohort of twelve patients with COVID-19 we can report that 6/12 (50%) of patients survived to hospital discharge, 5/6 had favourable neurological outcome. Due to the retrospective nature of our study, we are only able to report on patient survival till hospital discharge.

Although, ROSC rates hint a similar outcome we observed differences in rates to hospital discharge across the two study periods. This may be attributable to the location of cardiac arrest which shifted more towards the ICU in the 2020 period. Cardiac arrest occurring in the ICU shows clinically important differences compared to cardiac arrest occurring in a general ward [4, 5]. Due to this significant shift in location of cardiac arrest within the two study periods conclusion regarding outcome should be drawn cautious and ROSC may not present the most reliable indicator for survival to hospital discharge.
